# Phenotype and transcriptome analysis reveals chloroplast development and pigment biosynthesis together influenced the leaf color formation in mutants of *Anthurium andraeanum* ‘Sonate’

**DOI:** 10.3389/fpls.2015.00139

**Published:** 2015-03-11

**Authors:** Yuxia Yang, Xingxu Chen, Bin Xu, Yuxia Li, Yuehua Ma, Guangdong Wang

**Affiliations:** Department of Horticulture, Nanjing Agricultural UniversityNanjing, China

**Keywords:** *Anthurium andraeanum* Lind., leaf color mutants, transcriptome, chloroplasts, chlorophyll, pigment

## Abstract

Leaf color is one of the well-sought traits in breeding program for *Anthurium andraeanum* Lind. Knowledge of mechanisms in anthuriums to produce leaves with different shades of green would help to effectively select desirable traits. In this study, the micro- and ultra-structural and physiological features of leaves on wild type and leaf color mutants (*dark green, rubescent, etiolated, albino*) in *A. andraeanum* ‘Sonate’ were analyzed. Results show that chloroplasts of leaf color mutants exhibited abnormal morphology and distribution. Using next generation sequencing technology followed by *de novo* assembly, leaf transcriptomes comprising of 41,017 unigenes with an average sequence length of 768 bp were produced from wild type and *rubescent* mutant. From the 27,539 (67.1%) unigenes with annotated functions, 858 significantly differently expressed genes (DEGs) were identified, consisting of 446 up-regulated genes and 412 down-regulated genes. Genes that affect chloroplasts development and division, and chlorophyll biosynthesis were included in the down-regulated DEGs. Quantitative real-time PCR (qRT-PCR) analysis validated that the expression level of those genes was significantly lower in the *rubescent, etiolated*, and *albino* mutant compared to wild type plants, which concurs with the differences in micro- and ultra-structures and physiological features between these two types of plants. Conclusively, the leaf color formation is greatly affected by the activity of chloroplast development and pigment biosynthesis. And the possible formation pathway of leaf color mutant of *A. andraeanum* ‘Sonate’ is deduced based on our results.

## Introduction

*Anthurium andraeanum* Lind. is one of the most popular tropical evergreen perennial flowers because of its brilliant color and fantastic spathe shape. At present, improving the spathe size and color, leaf shape and color, and plant shape are goals for breeding of *A. andraeanum*. The development of an effective approach to obtain plants with beautiful leaves or flower color would require the basic knowledge of the molecular mechanism underlying color formation.

The contents of chlorophyll and anthocyanin are the primary factors influencing leaf color formation. While several pigments are produced in chloroplasts, chlorophyll is the major component in normal green leaves. Chloroplast is composed of chloroplast membrane, thylakoid and matrix. The number, shape and distribution of chloroplasts in leaf directly affect its color. Thus, dysfunctional chloroplasts usually result in leaf losing the green color. The leaf color of mutants is determined by the expression level of key genes involved in chlorophyll biosynthesis, chloroplast development and chloroplast division (Han et al., 1992; Eckhardt et al., 2004; Kato et al., 2007). Those genes directly or indirectly regulate the structure of chloroplasts, chlorophyll biosynthesis and several metabolic processes that affect the depth of leaf color (Jung et al., 2003; Motohashi et al., 2003; Chen et al., 2005). Several genes affecting chloroplast development and division have been found and their role in leaf color formation has been identified through researches on leaf mutants in *Arabidopsis thaliana*, rice (*Oryza sativa*), maize (*Zay mays*), and tobacco (*Nicotiana tabacum*). *GLK* (*Golden 2-like*) gene family includes *GLK1* and *GLK2*, which acts as regulation factors to regulate chloroplast development in diverse plant species, including maize, rice and *A. thaliana* (Langdale and Kidner, 1994; Hall et al., 1998; Rossini et al., 2001; Fitter et al., 2002; Yasumura et al., 2005). *Arabidopsis Pseudo Resoibse Regulator2* (*APRR2) is* a gene related but distinct from *the gene GLK2 in* tomato (*Solanum lycopersicum*). *GLK2* can increase plastid number, area, and pigment content when overexpressing in transgenic plants (Pan et al., 2013). *Accumulation and Replication of Chloroplast (ARC*) gene family participates in controlling the division of chloroplasts. Among this gene family, *ARC*3 (Gao et al., 2003), *ARC*5 (Shimada et al., 2004), and *ARC*6 (Vitha et al., 2003) are main regulation factors. Recent studies show that *ARC* genes cooperate with *FtsZ* to regulate chloroplast division (Osteryoung and Nunnari, 2003; Maple and Møller, 2007).

Flower color is determined by the accumulation of pigments such as carotenoids, betalains and anthocyanins (Chen et al., 2011). The anthocyanin biosynthetic pathway that determines floral pigmentation is generally conserved among different plant species, and genes related to this pathway have been isolated and extensively studied (Holton and Cornish, 1995; Tsuda et al., 2004; Koes et al., 2005). The expression patterns of genes responsible for anthocyanin biosynthesis, including *AaCHS* (chalcone synthase), *AaF3H* (flavanone 3-hydroxylase)*, AaDFR* (dihydroflavonol 4-reductase), and *AaANS* (anthocyanidin synthase), vary greatly among different tissues of *A. andraeanum* (Vern et al., 2004). However, the regulation pattern of anthocyanin biosynthesis in *A. andraeanum* not only differs from other ornamental systems studied to date but also does not match those characterized for regulating anthocyanin pigmentation in vegetative tissues in *A*. *thaliana* and *Perilla frutescens* (Shirley et al., 1995; Pelletier and Shirley, 1996; Gong et al., 1997; Vern et al., 2004). Besides, genes related to anthocyanin biosynthesis are commonly regulated by the MBW (MYB-bHLH-WD40) transcription complex (Feller et al., 2011; Hichri et al., 2011). Flower color is also affected by anthocyanin transport, but the mechanisms of anthocyanin transport from cytoplasm to the vacuole are unclear (Pourcel et al., 2010). The accepted hypothesis include the transport process mediated by GST (glutathione S-transferase) (Marrs et al., 1995), MRP (multidrug resistance- associated protein in the ABC transporter family) (Goodman et al., 2004), and vesicles and MATE (multidrug and toxic compound extrusion) (Gomez et al., 2009).

The number and size of chloroplasts are relatively stable in specific plant tissues, but they are apt to be influenced by genetic and environmental conditions (Mullet, 1993). Similarly, the biosynthesis and accumulation of pigments in flower are regulated by intrinsic and extrinsic factors. Most of the affected plants (mutants) might show abnormal leaf and flower color, meanwhile displaying changes in gene expression pattern. Investigating such mutants is helpful to explore the molecular mechanisms of leaf and flower color formation.

We have obtained leaf color mutants from a population of tissue culture-derived plantlets in *A. andraeanum* ‘Sonate’ (Xu et al., 2006). In this study, the micro- and ultra-structural and physiological characters of the mutants were further analyzed. Leaf transcriptomes from wild type and mutant plants were sequenced and differently expressed genes in the two groups of tissues were identified. The expression of genes involved in leaf color formation was validated by quantitative reverse transcription polymerase chain reaction (qRT-PCR). The main purpose of our study was to fully understand the gene expression difference between the wild type and the leaf color mutants, and explore the molecular mechanisms regulating pigment metabolism and leaf color mutant formation with the final aim of breeding desirable traits for color variation in *A. andraeanum*. The transcriptome database provides a valuable resource for genetic and genomic studies on leaf color formation in *A. andraeanum* and other plant species.

## Materials and methods

### Plant materials

**Figure 1:**
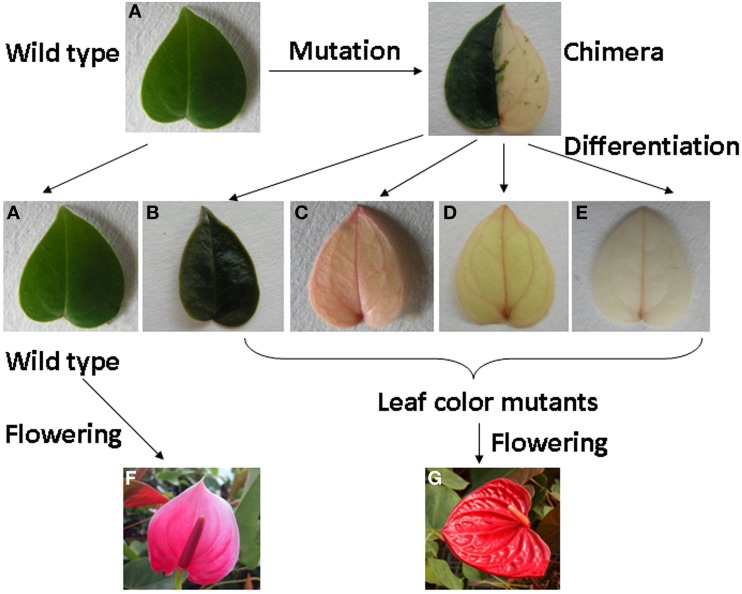
**The process of leaf color mutants formation and phenotype of wild type and leaf color mutants of *Anthurium andraeanum* ‘Sonate.’** **(A)** Wild type; **(B)** *dark green*; **(C)** *rubescent*; **(D)** *etiolated*; **(E)** *albino*; **(F)** Flower of wild type; **(G)** Flower of leaf color mutant.

The wild type and leaf color mutants, green type (*the dark green)* and non-green type (*rubescent, etiolated* and *albino*) (Figures 1A–E) of *A. andraeanum* ‘Sonate’ were used as the experimental materials. The 4 leaf color mutants were derived from an original mutant bearing half yellow and half green leaves. All of wild plants and leaf color mutants in pots were growing in the greenhouse at 25°C/20°C (day/night) without supplemental light at Nanjing Agricultural University, China for about 1 year. The *in vitro* plantlets incubated under a constant temperature of 25°C were used for micro- and ultra-structural and physiological studies. Newly emerged leaves from the mature wild and the *rubescent* plants were collected for transcriptome analysis. For qRT-PCR analysis, newly emerged leaves were collected from the mature wild and leaf color mutant plants growing in pots, and spathe tissues (when the spathe tightly furled) were harvested from mature wild and *dark green* plants. Immediately after harvest, samples were frozen in liquid nitrogen and stored at −80°C until analysis.

### Preparation of leaf and petiole sections

Transverse sections of leave and petiole were prepared by free-hand sectioning. The structure and distribution of chloroplasts on the transverse leaf and petiole sections were then observed under a light microscope (Leica DM1000, Leica Microsystems, Ltd, Germany).

### Isolation of protoplast

Protoplasts from leaf mesophyll cells of the wild type *Anthurium* and the 4 leaf color mutants were isolated following the method of Duquenne et al. (2007). The digest was passed through a 100 μm nylon sieve, and the filtrate was collected and observed under a fluorescence microscope (Zeiss Axioskop 40, Carl Zeiss, Germany). One hundred intact protoplasts per sample were observed for measuring the diameter of protoplast. The number of intact chloroplasts in the protoplasts was recorded.

### Transmission electron microscopy

Leaf discs of 1.0 × 2.0 mm in size were prepared from mature leaves dissected from wild type and mutant plants. After a pre-fixation procedure in 4% glutaraldehyde for 24 h at 4°C followed by 1% OsO_4_ for 2 h, tissues were dehydrated through an acetone series. Samples were embedded in Epon 812 and sectioned using a Leica ultramicrotome (Leica Microsystems, Ltd, Germany). After stained in 0.2% lead citrate, the ultrastructure of leaf cells was examined under a Hitachi H-7650 transmission electron microscope (Hitachi Science Systems, Ltd. Japan).

### Assays of contents of chlorophyll, carotenoid, and anthocyanin

About 0.1 g mature leaves from wild type and mutant plants were cut into pieces and then submerged in 5 ml acetone overnight, respectively. Then chloroplast pigments in the leaf extract were measured by specific light absorption at 665, 649, and 470 nm using a UV-1800 spectrophotometer (Shanghai Mapada Instruments Co. Ltd. China) (Arnon, 1949; Zhang, 1985). Following the procedure of Lewis et al. (2003) and Wu and Prior (2005), extract mixture (methanol: water: acetic acid = 85:15:0.5) was used to extract anthocyanin from leaves and petioles of the wild type and the 4 leaf color mutants, and spathes (when the spathe tightly furled) of wild type and *dark green* mutants. Three biological replicates were included for each sample. Obtained data was analyzed by LSD using the SPSS 10.0 program (SPSS Inc., Chicago, USA). Letters following values listed in analyzed tables indicated statistical differences (*p* ≤ 0.05) of contents of chlorophyll, carotenoid or anthocyanin between different types of plants.

### RNA extraction and preparation of cDNA library

Newly emerged leaves from wild type and *rubescent* mutants were collected and total RNA was isolated using the cetyltrimethylammonium bromide (CTAB) method (Wang et al., 2011). Two cDNA libraries each for the wild type and *rubescent* mutant were prepared for 100 bp paired-end RNA-Seq transcriptome. cDNA library construction and sequencing analysis were performed by Hangzhou Woosen Biotechnology Co. Ltd (Hangzhou, Zhejiang, China).

Poly (A) mRNA was purified with oligo (dT) beads from total RNA, and then the mRNA-enriched RNA was randomly segmented into 200–700 nt fragments in a divalent cation fragmentation buffer (Illumina, Hayward, CA) for 8 min at 94°C. These short fragments were used as templates to synthesize the first-strand cDNA using random hexamer primers and the second-strand cDNA was generated using RNaseH and DNA polymerase I. Those short cDNA fragments were purified with QiaQuick PCR extraction kit and washed with elution buffer (EB) for end repairing and tailing-A. Then those short fragments were ligated to sequencing adapters according to Illumina's protocol (San Diego, CA, USA) and further separated by agarose gel electrophoresis. Fragments of 300–500 bp were enriched by PCR amplification to create a cDNA library.

### Illumina deep-sequencing and *de novo* assembly

cDNA libraries were sequenced using the Illumina HiSeq™ 2000 sequencing system. Adaptor sequences were trimmed from raw reads, and reads containing “N” and low-quality reads (reads containing more than 50% bases with *Q* ≤ 20) were removed. Then *de novo* assembly was carried out using Trinity (Grabherr et al., 2011). Initially, the high quality reads were assembled into contigs with certain length of overlap. Then those reads were mapped back to contigs. Paired-end reads were used to calculate the distance and relation among these contigs. Finally, those contigs were scaffolded to generate unigenes using Cap3/PriceTI (Huang and Madan, 1999).

### Identification of DEGs in wild type and rubescent mutants

To analyze the expression level of the assembled unigenes in the leaves of wild type and *rubescent Anthurium*, all the assembled unigenes expression was calculated using FPKM (fragments per kilobase of exon model per million mapped reads) and the RPKM (reads per kb per million reads) methods in the ERANGE3.1 software (Mortazavi et al., 2008). Based on the FPKM and RPKM counts, all the assembled unigenes were classified into expressed genes (FPKM > 0), specifically expressed genes and differentially expressed genes (DEGs). The two libraries were analyzed by IDEG6 software to identify DEGs (Romualdi et al., 2003). FDR (the false discovery rate) was used to determine the threshold *P*-value in multiple tests. A DEG gene must have a FDR *P* < 0.001 and |log2 ratio| >1 which equals to a greater than two-fold difference in the number of transcripts from one sample to the other counterpart.

### Functional annotation

All of the unigenes were firstly compared to the NR database using the BLASTx analysis with a cut-off *E*-value of 10^−5^. And the GO, COG and the KEGG pathway annotation were also performed. The best alignments were used to determine sequence direction and to predict the coding regions of the assembled unigenes. A priority order of NR, KEGG and COG was followed if the same contig was not predicated to the same gene using different databases. ESTScan software (Iseli et al., 1999) was used to determine the orientation of unigenes when the unigene could not be aligned to any entries in these databases. The BLAST2GO software was used to analyze the GO annotations of unigenes (Conesa et al., 2005). Contigs in different GO term groups were analyzed using WEGO software (Ye et al., 2006) to reveal the overall distribution of gene functions. Pathway annotation for unigenes is obtained by Blastall software against the KEGG database (Kanehisa and Goto, 2000; Rismani et al., 2011). Then gene names or products, protein domains and GO terms were assigned to *Anthurium* transcripts according to the similarity to functionally characterized proteins or functional domains (Su et al., 2011). DEGs were also annotated with GO, COG and KEGG assignments. During the annotation process, *P* ≤ 0.05 and *Q* ≤ 0.05 were assigned as the threshold values for significant difference of gene expression in GO terms and KEGG pathways, respectively. At last, GOSeq software and KOBAS software were used to cluster gene expression patterns into statistically significantly enriched GO and KEGG pathways.

### Quantitative real-time PCR (qRT-PCR) analysis

Three sets of tissue samples were harvested for leaf from the wild type and leaf color mutants, and spathe from the wild type and *dark green* mutants. Total RNAs were extracted using the CTAB method. Removal of genomic DNA and the synthesis of first strand cDNAs were carried out following the manufactor's instruction (PrimeScript RT reagent kit With gDNA Eraser; Takara Bio Inc., Japan). Gene-specific primers (Table S1) for *AaGLK* (Unigene005017)*, AaARC5* (Unigene022169)*, AaMinE* (Unigene027030)*, AaAPRR2* (Unigene028254)*, AaDFR* (Unigene015437), *AaCHS* (Unigene035346)*, AaF3H* (Unigene026725)*, AaHEMC* (Unigene019324), *AaCRD1* (Unigene026313), and *AaPORC* (Unigene000637) were designed in Primer 5 using the assembled transcript sequence for each gene as the template. Among these unigenes, the expression style of *AaHEMC* (Unigene019324), *AaCRD1* (Unigene026313), and *AaPORC* (Unigene000637) were only analyzed in leaf from the wild type and 4 leaf color mutants because they regulate the chlorophyll biosynthesis. Glyceraldehyde-3-phosphate dehydrogenase (*AaGAPDH*, GenBank: JN602203.1) gene was selected as an internal reference gene.

The qRT-PCR reaction mixtures were prepared following the instruction in the SYBR® *Premix ExTag*™II reagent kit (Takara Bio Inc.). PCR reactions were performed on an ABI 7500 Real-Time PCR Detection System (Applied Biosystems, USA). The relative expression level of each unigene was normalized to *AaGADPH*. Three replicates of qPCR assay were performed for each gene. Data was analyzed using the Bio-Rad CFX Manager software and statistical test was analyzed by LSD using the SPSS 10.0 program (SPSS Inc., Chicago, USA). Different letters on the figure between the different types indicate their statistically difference at *P* ≤ 0.05.

## Results

### Phenotypes of the leaf color mutants

We have obtained 4 leaf color mutants in *A. andraeanum* ‘Sonate’ through indirect shoot regeneration from callus culture: *dark green* (Figure 1B), *rubescent* (Figure 1C), *etiolated* (Figure 1D), and *albino* mutants (Figure 1E). All 4 mutants were derived from an original mutant bearing half yellow and half green leaves. The *dark green* derived from the green half and the *rubescent, etiolated*, and *albino* mutants were all derived from the yellow half. The *dark green* leaf was darker, thicker and more rounded in shape while leaves from *rubescent, etiolated* and *albino* mutants were thinner than those of the wild type. The petiole and spathe of the mutants were red colored (Figure 1G), while those of wild type showed green and pink color respectively (Figure 1F). After long-term cultivation, phenotype of the *rubescent* was able to transform into *etiolated* and *albino* under changing environmental and nutrient conditions during the cultivation period, suggesting that the leaf color of these mutants might be regulated by extrinsic factors.

## Phenotypes in the properties of chloroplasts from leaf color mutants

### Chloroplasts distribution in leaves and petioles

**Figure 2:**
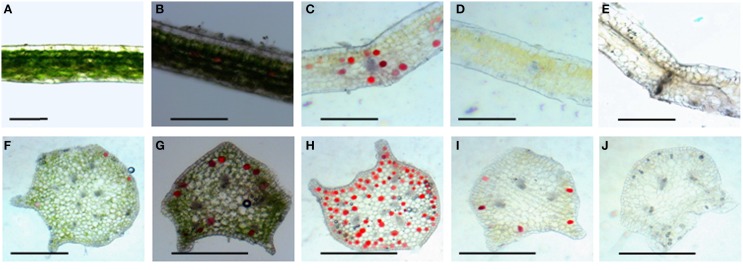
**Tissue configuration and chloroplast distribution in leaves and petioles of *Anthurium andraeanum* ‘Sonate.’ (A–E)** Transverse section of leaf tissue configuration and chloroplast distribution in leaf of Wild type, *dark green, rubescent, etiolated*, and *albino* mutants (Bar = 0.3 mm); **(F–J)** transverse section of petiole tissue configuration and chloroplast distribution in petiole of Wild type, *dark green, rubescent, etiolated*, and *albino* mutants (Bar = 0.2 mm).

Leaf color is controlled by the number and distribution of chloroplasts in the tissue. Results from hematoxylin-eosin staining showed that in wild type, chloroplasts densely was distributed in various layers of palisade and spongy mesophyll tissues, and a few chloroplasts existed in the stomata cells in the epidermis (Figure 2A). The 4 mutants did not display obvious differences in leaf structures (Figures 2B–E). The chloroplast distribution in leaves of the *dark green* mutant was similar to that of wild type, which could develop normally. However, very few chloroplasts were observed in leaf tissues of *rubescent, etiolated*, and *albino*, except that some stomata cells were distributed with relatively rich chloroplasts (Figures 2B–E). Surprisingly chloroplast distribution pattern in petioles of all 4 mutants was similar to the wild type (Figures 2G–J).

### Chloroplast morphology in leaf mesophyll cells

**Figure 3:**
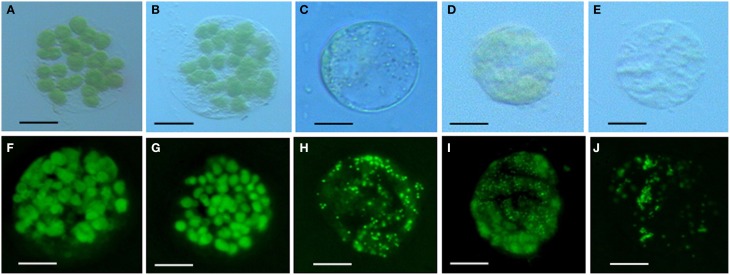
**Chloroplastic figures and chlorophyll spontaneous fluorescence in 5 types of mesophyllic cells. (A–E)** Single protoplast of mesophyllic cell of Wild type, *dark green, rubescent, etiolated*, and *albino* mutants; **(F–J)** chlorophyll spontaneous fluorescence of single protoplast of 5 types of mesophyllic cells. (Bar = 15 μm).

Chlorophyll in chloroplasts can emit auto fluorescence, thus the shape and quantity of chloroplasts could be measured respectively by the sizes of fluorescence dots and fluorescence density (Figure 3). Our data showed that isolated protoplasts were round in shape, varied in sizes, and contained different amounts of chloroplasts (Table 1). Notably, in the *rubescent, etiolated*, and *albino*, very few intact chloroplasts, but dot- or mist-like plastids were observed (Figures 3C–E,H–J). There were dot-like plastids in the mesophyll cells of *rubescent* (Figures 3C,H). Small “nebular” plastids presented in mesophyll cells of *etiolated* (Figures 3D,I). Almost transparent yellow plastids with sporadic fluorescent dots were observed in mesophyll cells of *albino* (Figures 3E,J).

**Table 1:** **Diameters and quantities of intact chloroplasts per protoplast in the wild type and mutants of *A*. *andraeanum* ‘Sonate’**.

**Types of leaf**	**Diameters (μm)**	**Quantities of intact chloroplasts per protoplast (Entries)**
Wild type	40–45	35–45
*dark green*	40–45	40–50
*rubescent*	35–40	Intact chloroplasts appeared sporadically
*etiolated*	25–30	Intact chloroplasts appeared sporadically
*albino*	25–30	Intact chloroplasts appeared sporadically

### Chloroplast ultrastructure

**Figure 4:**
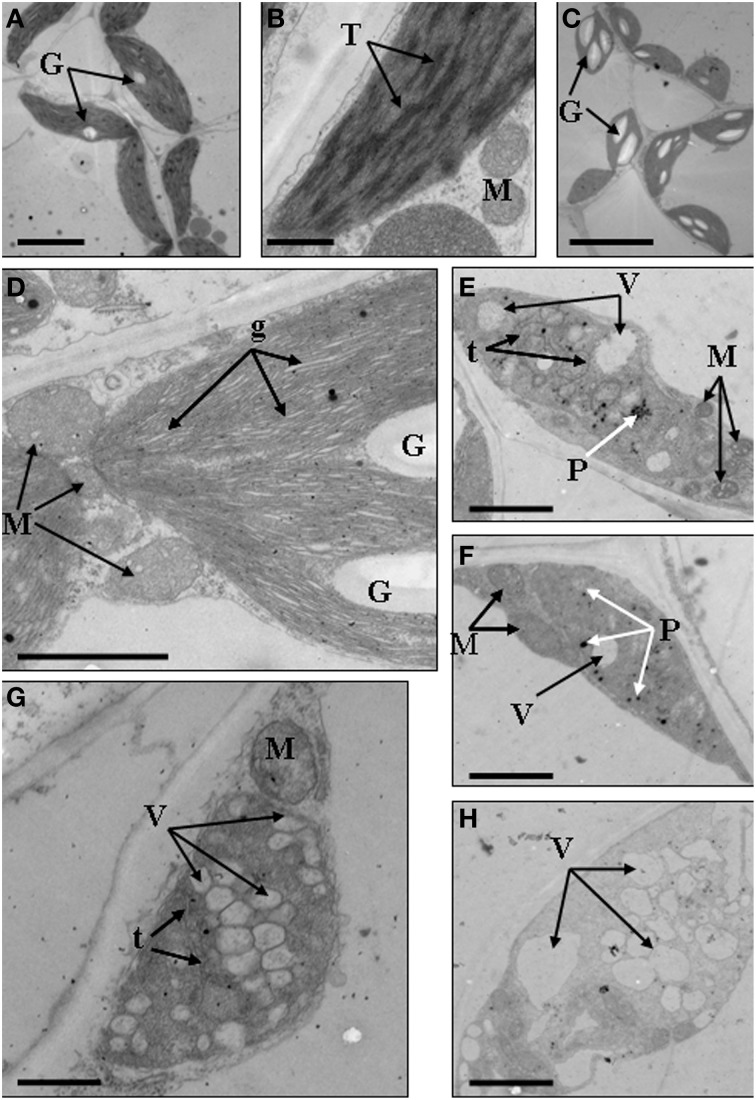
**Chloroplast ultrastructures of wild type, *dark green, rubescent, etiolated* and *albino* mutants of *Anthurium andraeanum* ‘Sonate’**. **(A,B)** Chloroplast ultrastructures of Wild type (In **A**, Bar = 5 μm; in **B**, Bar = 0.5 μm); **(C,D)** Chloroplast ultrastructures of *dark green* mutant (In **C**, Bar = 10 μm; in **D**, Bar = 1 μm); **(E,F)** 2 different chloroplast ultrastructures of *rubescent* mutant (Bar = 1 μm); **(G)** chloroplast ultrastructures of *etiolated* mutant (Bar = 1 μm); **(H)** chloroplast ultrastructure of *albino* mutants (Bar = 1 μm). In these pictures, G, denotes granulose; T, denotes thylakoid grana; M, denotes mitochondria; g, denotes gaps between grana lamellas; t, denotes single thylakoid sac; P, denotes plastoglobuli; V, denotes vacuole.

We further analyzed the ultrastructure of chloroplasts to confirm the abnormal development of chloroplasts. Results showed that in the mesophyll cells of wild type, chloroplasts showed typical structures, containing small starch granules and a few plastoglobuli (Figures 4A,B). In the *dark green* mutant, chloroplasts were inflated because of the accumulation of large starch granules, which also led to the formation of big gaps between stroma thylakoids (Figures 4C,D). In the *rubescent* mutant, chloroplasts did not have intact thylakoid structures, and some chloroplasts contained irregularly arranged vesicles (Figures 4E,F), meanwhile some chloroplasts were encompassed by mitochondria (Figure 4E). In the *etiolated* mutant, chloroplasts were filled with vesicles. Few of vesicles retained the thylakoid membrane, but they were not real plastoglobulus (Figure 4G). Mitochondria were also observed around some of the chloroplasts (data not shown). In the *albino* mutant, chloroplasts were crowded by vesicles which had almost no inner member structures. Some of the chloroplasts were also surrounded by mitochondria (Figure 4H). In summary, the ultrastructure of chloroplasts in mutant leaves were disrupted, confirming that the chloroplast development was impaired.

### Content of chlorophyll, carotenoid, and anthocyanin

Leaf color is determined by the content of chlorophyll and other pigments accumulated in the tissue. Our data indicated that contents of chlorophyll and carotenoid were significantly different between the wild type and the 4 mutants (Table 2). The contents of chlorophyll and carotenoid from high to low were in the leaf of *dark green*, wild type, *etiolated, rubescent*, and *albino*. The ratio of chl a/chl b in *dark green, rubescent*, and *etiolated* mutants was higher than those in the wild type. The ratio of carotenoid/total Chl in all 4 mutants was higher than that of the wild type. Furthermore, the content of anthocyanin in the leaf and petiole of *dark green* and *rubescent* mutants was higher than that of the wild type (Table 3). The content of anthocyanin was at a similar level in the leaf and petiole of *etiolated* mutant and wild type plants (Table 3). The *albino* leaves had the lowest anthocyanin content (Table 3). Consistently, the spathe of the *dark green* mutant had a higher content of anthocyanin than the wild type plants (Table 3). Our data suggest that the abnormal leaf and spathe color of mutants was caused by changes in pigment compositions.

**Table 2:** **Content of chlorophyll and carotenoid in the wild type and mutants leaves in *A*. *andraeanum* ‘Sonate’**.

**Plants**	**Chl a (mg·g^−1^)**	**Chl b (mg·g^−1^)**	**Chl a + b (mg·g^−1^)**	**Chl a/b**	**Carotenoid (mg·g^−1^)**	**Carotenoid/Chl**
Wild type	1.285 ± 0.037b	0.432 ± 0.017a	1.718 ± 0.021a	2.970d	0.304 ± 0.045b	0.177e
*dark green*	1.502 ± 0.042a	0.333 ± 0.065b	1.836 ± 0.057a	4.508a	0.566 ± 0.055a	0.308c
*rubescent*	0.019 ± 0.004c	0.006 ± 0.001c	0.025 ± 0.004b	3.407c	0.019 ± 0.005c	0.767a
*etiolated*	0.056 ± 0.009c	0.013 ± 0.001c	0.069 ± 0.008b	4.203b	0.028 ± 0.010c	0.401b
*albino*	0.010 ± 0.004c	0.006 ± 0.004c	0.016 ± 0.001b	1.716e	0.003 ± 0.001c	0.206d

*Different letters within the same column indicate significant difference by LSD's multiple range test at 5% level*.

**Table 3:** **Content of anthocyanin in the wild type and mutants in *A. andraeanum* ‘Sonate’**.

**Plants**	**Leaf (Anthocyanin mg·g^−1^)**	**Petiole (Anthocyanin mg·g^−1^)**	**Spathe (Anthocyanin mg·g^−1^)**
Wild type	0.049 ± 0.002c	0.053 ± 0.001c	0.097 ± 0.001b
*dark green*	0.151 ± 0.003b	0.175 ± 0.011a	0.378 ± 0.026a
*rubescent*	0.179 ± 0.001a	0.150 ± 0.001b	-----------------
*etiolated*	0.047 ± 0.001c	0.045 ± 0.001c	-----------------
*albino*	0.011 ± 0.002d	0.015 ± 0.004d	-----------------

*Different letters within the same column indicate significant difference by LSD's multiple range test at 5% level*.

## Transcriptome analysis

### Illumina sequencing and *de novo* assembly

**Figure 5:**
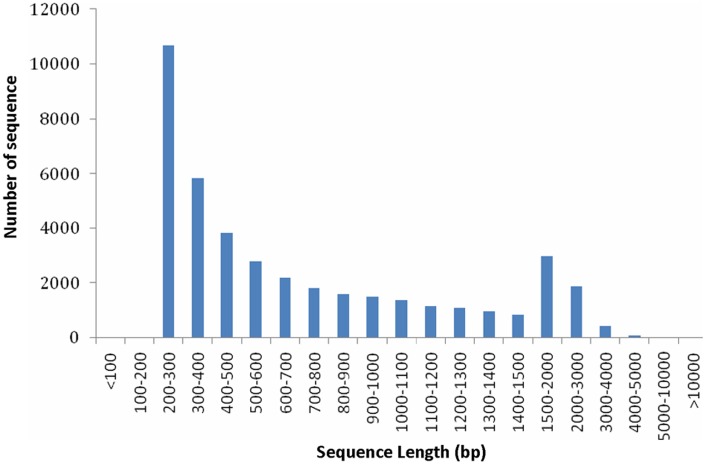
**Length distributions of unigenes**.

From the above described micro- and ultra-morphology and physiological features, we proposed that the expression pattern of genes responsible for chloroplast development and division and pigment biosynthesis may have been altered in mutants. To testify our hypothesis, transcriptome comparison was carried out. Because the 4 leaf color mutants came from an original mutant, we supposed they should share the same mechanism in leaf color formation, thus each of them could represent all of them. The *dark green* was more complex: it had chloroplast with normal function meanwhile showed characteristics of mutants. In contrast, the other 3 mutants (*rubescent, etiolated*, and *albino*) displayed similar and relatively simple phenotype (but showed difference in mutation degree: weak, moderate and severe, respectively). Thus, here *rubescent* was selected as the representative mutant to compared with the wild type to explore the mechanism of the leaf color mutation based on transcriptome analysis. As a result, approximately 4 Gbp of sequence data was produced. A total of 39,119,350 and 45,807,980 raw reads were obtained respectively by sequencing the cDNA libraries prepared from the wild type and *rubescent* mutant leaves. After trimming of the adapter sequences, and removal of low quality reads with N greater than 5%, the remaining 39,107,374 clean paired-end reads with 3,790,089,187 nucleotides in wild type library, and 45,794,218 paired-end reads with 4,445,749,574 nucleotides in *rubescent* mutant library, were generated, respectively. Using *de novo* assembly, 48,284 contigs consisting of 37,289,226 nucleotides were produced, with an average length of 772 bp and N50 (represents median length of all contigs) of 1155 bp. The length distribution of the assembled contigs was shown in Figure S1. At last, all of the assembled contigs were further scaffolded into 41,017 unigenes with a N50 length of 1172 bp and the mean unigene size was 768 bp (Table S2). The majority (50.3%) of the unigenes were longer than 500 bp. The specific length distributions of unigenes were given in Figure 5. This Transcriptome Shotgun Assembly project has been deposited at DDBJ/EMBL/GenBank under the accession GBKP00000000.

### Analysis on differential gene expression

**Figure 6:**
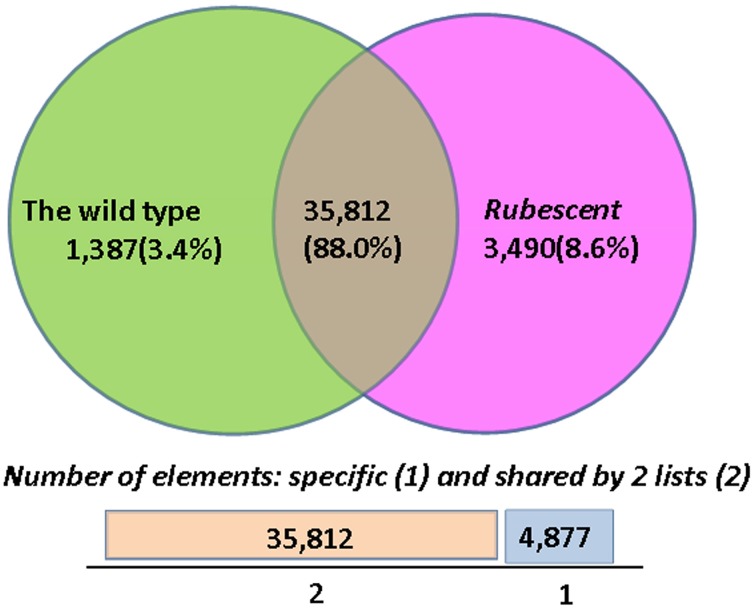
**The numbers of specific genes and shared genes between the wild type and *rubescent* mutants of *Anthurium andraeanum* ‘Sonate’**.

The FPKM and RPKM methods were used to analyze the gene expression in the two libraries. As a result, 37,199 and 39,302 unigenes were identified respectively in the cDNA library from wild type and *rubescent* mutant leaves, of which 1387 and 3490 genes expressed specifically in leaves of the wild type and *rubescen* mutant, respectively (Figure 6). When comparing the two libraries according to RPKM calculation, a total of 858 differently expressed genes (DEGs) were detected, including 446 up-regulated genes and 412 down-regulated genes. After analysis on all DEGs, we found that more than 50% of the DEGs did not have homologous sequences meeting the search criteria in the NCBI database. These unmatched unigene sequences may contain fragmented RNAs from untranslated regions (UTRs), long non-coding RNAs (ncRNAs) and other types of transcripts, or they are the specific genes in *A. andraeanum*.

### Functional annotation

All of the unigenes were searched in the GenBank's non-redundant protein database (NR), the Gene Ontology (GO), Clusters of Orthologous Groups (COG) and the Kyoto Encyclopedia of Genes and Genomes (KEGG) pathway databases. A total of 27,539 (67.1%) unigenes matched a sequence in at least one of the above public databases. Furthermore, there were 26,997 (65.8%) unigenes matching genes from model plant *A. thaliaba* and 26,946 (65.7%) unigenes matching genes in the *Oryza sativa* japonica group database. Finally, 30,419 (74.2%) unigenes were predicted to be coding genes (Table S3).

**Figure 7:**
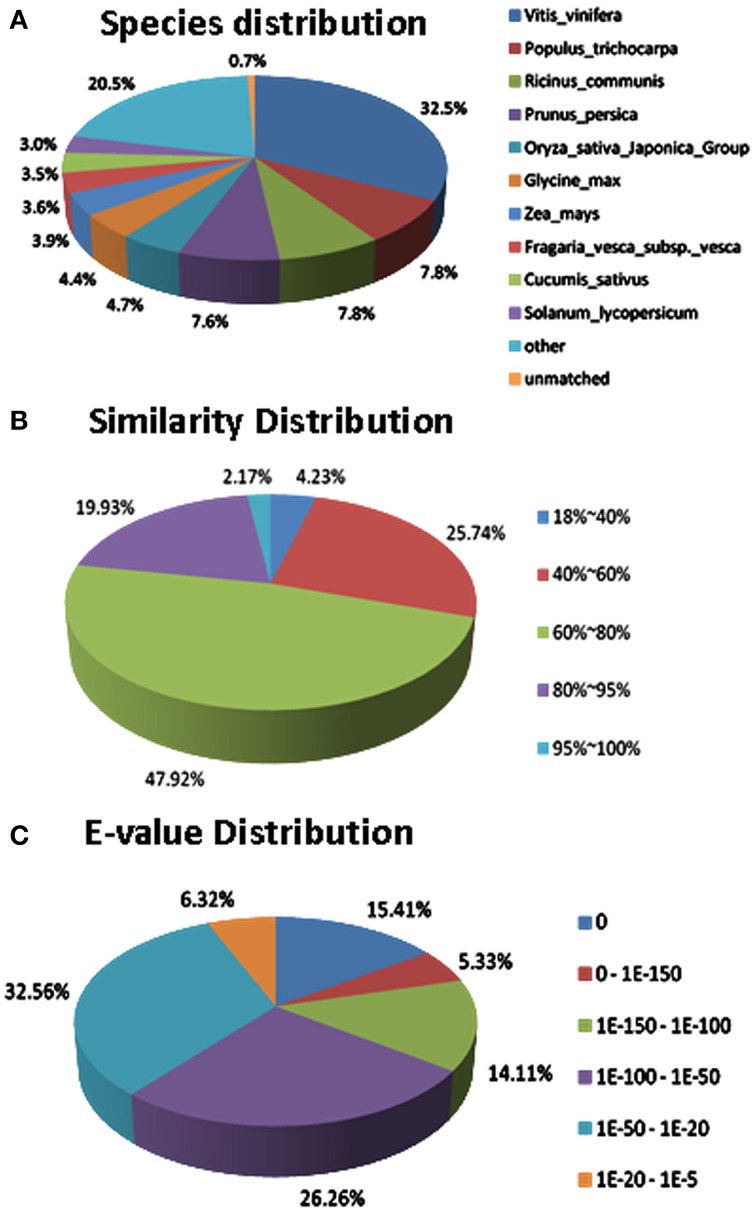
**Characteristics of sequence homology of *Anthurium* BLASTED against NCBI non-redundant (Nr) database**. **(A)** Species distribution of the top BLAST hits. **(B)** Similarity distribution of top BLAST hits for each unigene. **(C)** *E*-value distribution of BLAST hits for matched unigene sequences, using an *E*-value cutoff of 1.0E^−5^.

Regarding to the NR annotation, 25,456 (62.1%) unigenes has a hit in the database. Further analysis of the BLAST data showed that some of the annotated sequences share identical fragments with genes from *Vitis vinifera* (32.5%), *Populus trichocarpa* (7.8%), *Ricinus communis* (7.8%), and *Prunus persica* (7.6%) (Figure 7A). For similarity distribution, 70.0% of the sequences showed a similarity higher than 60%, while more than half of the annotated sequences had a similarity level ranging from 60 to 80% (Figure 7B). Furthermore, the *E*-value distribution pattern indicated that 61.1% of the top hits had high homology with the *E* <1.0e^−50^, and 38.8% of the annotated sequences showed a moderate homology with the *E*-value between 1.0e^−5^ and 1.0e^−50^ (Figure 7C).

The functions of All-unigenes and DEGs were classified according to GO database through Blast2GO suite. As a result, 11,593 (28.3%) All-unigenes and some DEGs were classified into three different categories: cellular component, molecular function and biological process (Figure S2). They could be further classified into 41 functional groups (Figure S2). We found that the GO terms of DEGs were exactly similar to the All-unigenes (Figure S2).

**Figure 8:**
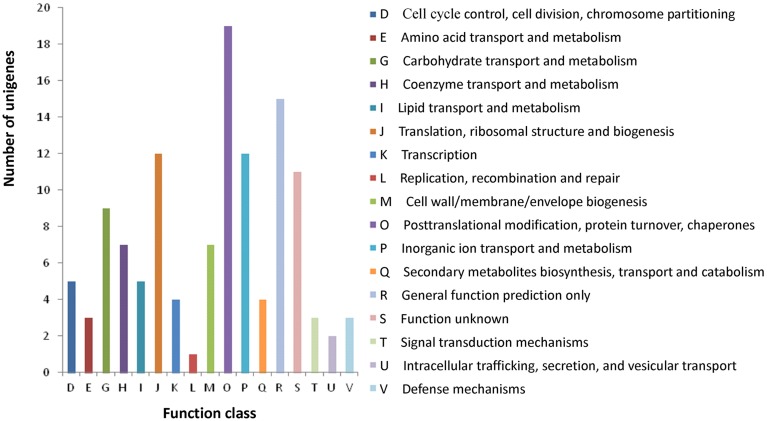
**COG classification of the annotated 102 DEGs**. The capital letters in x-axis indicate the COG categories as listed on the right of the histogram and those on y-axis indicate the number of DEGs.

All assembled unigenes were further annotated based on COG category to produce phylogenetic classification. In total, 10,701 (26.1%) unigenes were matched, and they were grouped into 24 functional categories (Figure S3). The main purpose of our study was to fully analyze the gene expression difference between the wild type and *rubescent*, so the analysis with COG focused on the DEGs. As a result, a total of 102 (11.9%) DEGs were assigned to 122 COG functional annotations, which were classified into 17 COG categories (Figure 8).

To identify the biological pathways activated in *A. andraeanum* ‘Sonate,’ all unigenes were annotated with KEGG Orthology (KO) numbers using BLASTx alignments against KEGG, using an *E*-value cut-off of 1.0E^−5^. As a result, a total of 5245 (12.8%) unigenes were significantly matched in KEGG pathway database, and were assigned to 261 KEGG pathways. The result showed that the “Metabolic pathways” was the greatest part (1734, 33.1%), followed by “Biosynthesis of secondary” (755, 14.4%) (Table 4). Furthermore, to understand the biological function of these DEGs, all the DEGs were also mapped to terms in KEGG database. Finally, 66 (7.7%) DEGs were matched and assigned to 161 KEGG pathways. Similar to the All-unigenes, the mainly enriched pathways of the DEGs were in “Metabolic pathways,” “Biosynthesis of secondary metabolites,” and several other clusters (Table 4).

**Table 4:** **Significantly enriched pathways of all unigenes and DEGs in *A*. *andraeanum* ‘Sonate’**.

**Pathway**	**No. of all genes with pathway annotation (5245)**	**No. of DEGs with pathway annotation (66)**		**Pathway ID**
		**Up-regulated genes**	**Down-regulat-ed genes**	
Metabolic pathways	1734 (33.1%)	13 (19.7%)	9 (13.6%)	Ko01100
Biosynthesis of secondary metabolites	755 (14.4%)	7 (10.6%)	8 (12.1%)	Ko01110
Microbial metabolism in diverse environments	400 (7.6%)	4 (6.1%)	2 (3.0%)	Ko01120
Ribosome	234 (4.5%)	1 (1.5%)	2 (3.0%)	Ko03010
Protein processing in endoplasmic reticulum	234 (4.5%)	9 (13.6%)	1 (1.5%)	Ko04141
RNA transport	227 (4.3%)	1 (1.5%)	–	Ko03013
Plant-pathogen interaction	221 (4.2%)	4 (6.1%)	4 (6.1%)	Ko04626
Plant hormone signal transduction	214 (4.1%)	–	3 (4.5%)	Ko04075
Aminoacyl-tRNA biosynthesis	91 (1.7%)	3 (4.5%)	–	Ko00970
Basal transcription factors	71 (1.4%)	1 (1.5%)	–	Ko03022
ABC transporters	36 (0.7%)	1 (1.5%)	–	Ko02010
Metabolism of xenobiotics by cytochrome P450	27 (0.5%)	2 (3.0%)	–	Ko00980
Flavonoid biosynthesis	23 (0.4%)	1 (1.5%)	–	Ko00941

### Analysis on genes related to chloroplast development and division, chlorophyll and anthocyanin biosynthesis

Based on the above annotations, we found that genes regulating chloroplast development and division, chlorophyll biosynthesis, pigment biosynthesis and transport were all included in the *A. andraeanum* ‘Sonate’ transcriptome database (Table 5). Further more, compared to wild type, the expression levels of *GLK* (chloroplast development), *Ftsz* and *MinE* (chloroplast division), *HEMB* (Hydroxymethylbilane synthase)*, CRD1* and *PORC* (chlorophyll biosynthesis), *ABC* and *MATE* (anthocyanin transport), *APRR2* (pigment biosynthesis) in *rubescent* mutant were down-regulated, suggesting that the decreased expression of these genes might contribute to the abnormal leaf color of *rubescent* mutant.

**Table 5:** **Unigenes and transcription factors involved in chlorophyll, anthocyanin biosynthesis in the *A*. *andraeanum* ‘Sonate’ transcriptome**.

**Function**	**TF family**	**Expression level in *Rubescent***	**Annotation**
Chloroplast development	GLK	Down-regulated	Transcription activator GLK1-like[*Vitis vinifera*]
Chloroplast division	Ftsz	Down-regulated	Tubulin/FtsZ family protein
	MinD	Down-regulated	Septum site-determining protein (MIND)
	MinE1	Up- regulated	Homolog of bacterial MinE1
Chlorophyll biosynthesis	HEMA1	Down-regulated	Glutamyl-tRNA reductase family protein
	GSA2	Up- regulated	Glutamate-1-semialdehyde 2,1-aminomutase 2
	HEMB1	Down-regulated	Aldolase superfamily protein
	HEMC	Down-regulated	Hydroxymethylbilane synthase
	HEMD	Down-regulated	Uroporphyrinogen-III synthase family protein
	HEME1	Down-regulated	Uroporphyrinogen decarboxylase
	HEMG2	Down-regulated	Flavin containing amine oxidoreductase family
	CHLD	Up- regulated	ALBINA 1
	GUN5	Down-regulated	Magnesium-chelatase subunit chlH, putative (CHLH)
	CHLI1	Up- regulated	P-loop containing nucleoside triphosphate hydrolases superfamily protein
	CHLM	Down-regulated	Magnesium-protoporphyrin IX methyltransferase
	CRD1	Down-regulated	Dicarboxylate diiron protein, putative (Crd1)
	PORA	Up- regulated	Protochlorophyllide oxidoreductase A
	PORB	Up- regulated	Protochlorophyllide oxidoreductase B
	PORC	Down-regulated	Protochlorophyllide oxidoreductase C
Anthocyanin transport	ABC	Down-regulated	ABC transporter family protein
	GST	Up- regulated	Glutathione S-transferase family protein
	MATE	Down-regulated	MATE efflux family protein
Pigment biosynthesis	APRR2	Down-regulated	Two-component response regulator-like APRR2[Vitis vinifera]
	CHS	Down-regulated	Chalcone and stilbene synthase family protein
	F3H	Up- regulated	flavanone 3-hydroxylase
	DFR	Up- regulated	dihydroflavonol 4-reductase
	UF3GT	Up- regulated	UDP-glucose:flavonoid 3-o-glucosyltransferase
Transcription factors	MYB	Down-regulated	MYB-CC type transfactor
	Bhlh	Down-regulated	Basic helix-loop-helix (bHLH) DNA-binding family protein
	WD40	Down-regulated	WD40 repeat-like superfamily protein
		Down-regulated	bHLH-MYC and R2R3-MYB transcription factors N-terminal

In summary, results on transcriptome analysis were consistent with the phenotypes of mutants and wild type plants. These results indicate the abnormal leaf color of mutants was caused by changes in the expression pattern of genes responsible for pigment biosynthesis.

### Validation and expression analysis of DEGs

**Figure 9:**
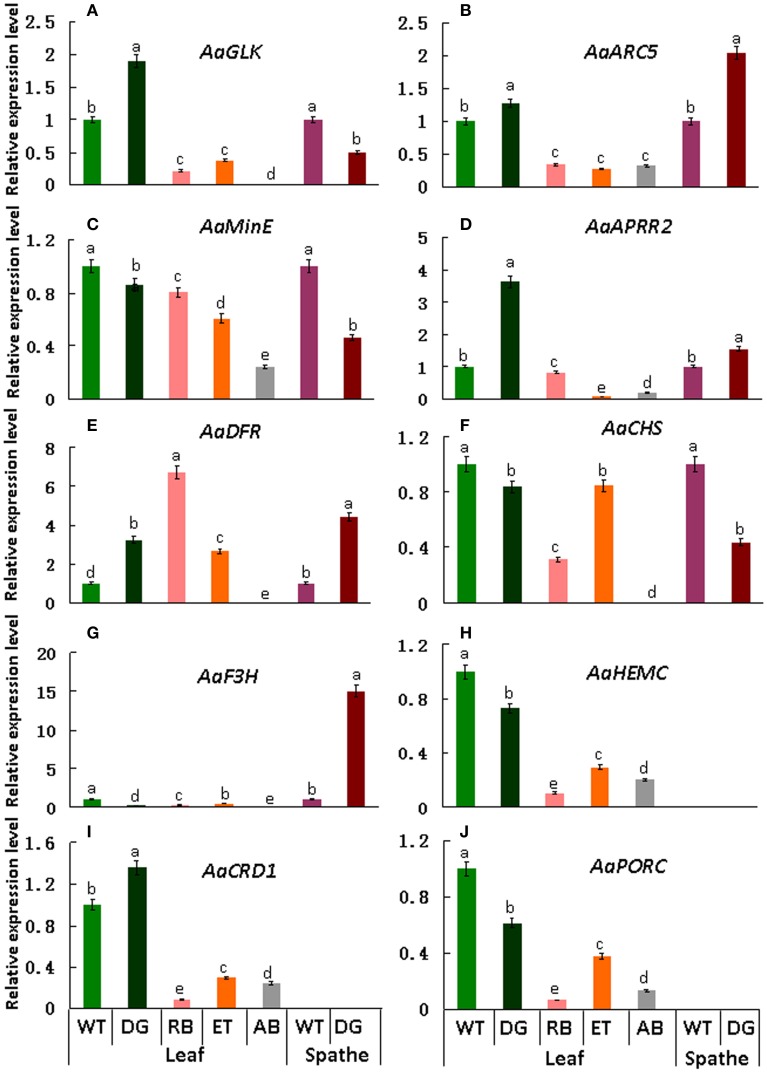
**qRT-PCR expression analysis of genes involved in chloroplasts development, division and pigment biosynthesis in different tissues in the wild type *Anthurium* and 4 leaf color mutants**. In these pictures, WT, Wild type; DG, *dark green*; RB, *rubescent*; ET, *etiolated*; AB, *albino*. **(A)** the relative expression level of *AaGLK;* **(B)** *AaARC5;* **(C)** *AaMinE;* **(D)** *AaAPRR2;* **(E)** *AaDFR;* **(F)** *AaCHS;* **(G)** *AaF3H;* **(H)** *AaHEMC;* **(I)** *AaCRD1;* **(J)** *AaPORC*. Different small letter with in lines indicate significant difference by LSD's multiple range test at 5% level in the leaves and spathes, respectively.

qRT-PCR was performed to validate the differential expression level of target genes in mutant and wild type plants. Results indicate that the transcript levels of *AaGLK, AaARC5*, and *AaAPRR2* in wild type were lower than that of *dark green* in the leaf, and higher than that of *rubescent, etiolated*, and *albino* in leaf (Figures 9A–D). Consistent with leaf color differences, the expression of *AaDFR* (anthocyanin biosynthesis) in wild type leaf was lower than that in *dark green, rubescent*, and *etiolated* mutants. And in spathe of *dark green* the expression of *AaDFR* was higher than that in the wild type (Figure 9E). Interestingly, *AaCHS* (chalcone and stilbene biosynthesis) showed a higher expression in tissues of wild type than that of *dark green* (leaf and spathe), *rubescent, etiolated, and albino* mutants (leaf) (Figure 9F). Similar to *AaCHS, AaF3H* (flavanone and anthocyanin biosynthesis) expression was down-regulated in the leaf of *4 leaf color mutants* but up-regulated in the spathe mutant (of *dark green*) (Figure 9G). *AaHEMC, AaCRD1* (Dicarboxylate diiron protein) and *AaPORC* (Protochlorophyllide oxidoreductase C) (chlorophyll biosynthesis) expressed a higher quantity in the leaf from the wild and dark green type than that in *rubescent, etiolated and albino* mutants (Figures 9H–J). Thus, the expression pattern of *AaCHS* and *AaF3H* reflects the complex process of color formation.

The qRT-PCR results basically validated the expression level of DEGs found in transcriptome database and were consistent with the phenotypes of leaf color mutants.

## Discussion

### Leaf color reflected the developmental characteristics of chloroplasts

Leaf color formation depends on the components of pigment accumulation in plastid, which involves in chloroplast development and division, chlorophyll biosynthesis and transport, and pigment biosynthesis. Thus, changes in leaf color might reflect the abnormal development and function of plastid. It is reported that lycopene associates with the remnant membrane system evolved from the chloroplast thylakoid grana, and β-carotene associates with the globular and rod- or tablet-shaped crystal structures in the chromoplast (Harris and Spurr, 1969a,b). Therefore, variation at the pigment accumulation might be accompanied with alteration in plastid structures because of the degradation of the highly structured thylakoid membrane system (Rosso, 1968). In our study, the structure and quantity of chloroplasts in leaf color mutants were significantly distinct from that of wild type (Table 1, Figures 2–4). And our data showed that the micro- and ultra-structures of chloroplasts from leaf color mutants were severely changed (Figures 2–4), suggesting a typical process of chloroplast-chromoplast transition (Cheung et al., 1993). Furthermore, the content of chlorophyll in the *rubescent* was significantly lower than that in the wild type and the *dark green* mutants (Table 2). Consistent with the leaf color (Figure 1), the content of anthocyanin in the *rubescent* and the *dark green* mutants was higher than that in the wild type (Table 3). In addition, no prolamellar body was seen in the plastids of *rubescent* and *etioplast* mutants under ultrastructure observation (Figures 4C,D). Those results demonstrated that the interruption of chlorophyll synthesis or transportation of protochlorophyllide oxidoreductase might have stalled the transformation process from etioplasts to chloroplasts.

### Sequencing and *de novo* assembly of the transcriptome from *A. andraeanum* ‘Sonate’

Transcriptomic profiling provides information about quantitative changes in gene expression, which helps to gain fundamental insights into biological processes, and reveal the temporal and spatial expression pattern of the whole genome (Wang et al., 2009). Moreover, RNA-seq discovers novel genes specific to certain species (Surget-Groba and Montoya-Burgos, 2010).

We speculate that in those leaf color mutants, changes in gene expression that are responsible for chloroplast development and division have occurred. In this study, using deep sequencing and *de novo* assembly approach, 41,017 unigenes were obtained for leaf transcriptomes, of which 10,783 (26.2%) unigenes are ≥1000 bp in length (Figure 7) and 20,667 (50.4%) unigenes are ≥500 bp (Figure 5). Most of these unigene sequences are greater than those generated in a previous sequencing project of the same species (*A. andraeanum)* (Tian et al., 2013). Our study indicates that a high-coverage transcriptome database can be produced by using a combination of short-read deep sequencing strategy and an effective sequence assembly tool. However, some key genes involved in the development and function of chloroplasts were still not captured, such as *ARTEMIS* (Fulgosi et al., 2002), *AtFZL* (Gao et al., 2006), *MSLs* (Haswell and Meyerowitz, 2006). These results suggest that deeper sequencing, or sequencing of subcellular transcriptomes instead of the whole leaf, may be needed in order to capture all the genes expressed in leaf tissues.

Most importantly, this is the first study to provide abundant transcriptome information for leaf color mutants in *A. andraeanum*. The unigene database will enable researchers to understand the structure and expression of genes relevant to leaf color formation in *A. andraeanum*. Finally, the transcript sequences can be used to develop molecular and cellular markers in breeding programs as well as future genome research, and the frequency of the mapped reads can be used to assess the expression level of genes.

### Analysis of differentially expressed genes (DEGs) in the transcriptome database

According to the transcriptome analysis, compared to the wild type, a total of 858 DEGs were identified in leaf color mutant of *rubescent*, including 446 up-regulated genes and 412 down-regulated genes. Consistent with our original prediction, leaves of mutant plants showed a differential expression level of genes regulating chloroplast development and division, chlorophyll biosynthesis, and pigment biosynthesis and transport, such as *GLK, FtsZ, MinD, HEMC, CRD1, ABC, APRR2, DF*R, and others. This indicates that these processes are affected thus leading to the development of abnormal leaf color in those leaf color mutants. Besides, we found that 12 DEGs (9.8%) were classified into “translation, ribosomal structure and biogenesis” under COG category. The reduced expression of chloroplast ribosomal genes can affect protein translation required for the biogenesis of the sub-organelles (Wallas et al., 2003).

### The expression level of genes participating in chloroplast development and division were altered in leaf color mutants

Normal development of chloroplasts of higher plants requires the coordination of chloroplast genes and nuclear genes. Alteration in the expression of either genome may affect the biogenesis of normal chloroplasts. The resultant disruption in chlorophyll metabolism and chloroplast assembly can lead to abnormal leaf color. Previous study suggested that *GLK* gene family acted as regulative factor for chloroplast development in maize, rice and *A. thaliana* (Langdale and Kidner, 1994; Hall et al., 1998; Rossini et al., 2001; Fitter et al., 2002; Yasumura et al., 2005; Waters et al., 2008, 2009). Leaves of maize *g2* (*golden2, Zmglk2*) mutants present golden color, in which chloroplasts are smaller than wild type and develop only rudimentary thylakoid lamellae that exhibit very few grana (Langdale and Kidner, 1994). In *A*. *thaliana*, analysis on insertion mutants of *Atglk*1 and *Atglk2* demonstrated that single mutant displays normal leaf color, reflecting some degree of functional redundancy. However, double mutants are pale green in all photosynthetic tissues and chloroplasts exhibit a reduction in granal thylakoids and smaller size in cross-sectional area than the wild type (Fitter et al., 2002; Waters et al., 2009). Consistent with these research findings, in our study the wild type and *dark green* plants developed mature chloroplasts compared to those in the *rubescent, etiolated and albino* mutants which did not have distinct thylakoid structures (Figures 2–3). Meanwhile, the expression level of *AaGLK* in leaves of wild type and *dark green* was higher than that in the *rubescent, etiolated and albino* mutants (Figure 9A). In conclusion, our research has further proven that the expression level of *GLK* gene is closely related with chloroplast development in various plant species.

The mechanism of chloroplast division has been studied deeply, and some members of division complex body have been identified in the *ARC* gene family. Previous researches demonstrated that *ARC*3 (Gao et al., 2003), *ARC*5 (Shimada et al., 2004), and *ARC*6 (Vitha et al., 2003) are the main regulation factors. Mesophyll cells in *A. thaliana* mutant *arc3* have a small number of abnormal and large chloroplasts (Gao et al., 2003), mutants of *arc5* have enlarged, dumbbell-shaped chloroplasts (Shimada et al., 2004), and mutants of *arc6* contain only one or two grossly enlarged chloroplasts (Vitha et al., 2003). Our study showed that the chloroplasts in the wild type and *dark green* were bigger than those in the *rubescent, etiolated and albino* mutants, and furthermore the former two plants contained more intact chloroplasts than the latter mutants which are similar to the phenotype of chloroplasts in *arc5* mutant (Table 1). Interestingly, the expression level of *AaARC5* in the wild type and 4 leaf color mutants were in accordance with the chloroplasts morphology (Figure 9B). Our data also indicated there were very few chloroplasts in the mesophyll cells of *rubescent* mutant (Table 1). And those small chloroplasts showed dot-like shape, they were far smaller than those in the other three mutants even though some of them contained rudimentary thylakoids (Figures 3B–E). We have isolated chloroplasts that are similar in morphology to that of *arc5* mutant (Figure 4F), but the chloroplast number was far lower than that of *arc5*. As it is known, *GLK* and *ARC5* respectively controls the number and structure of chloroplasts, and these two genes have no direct influence upon the function of each other (Pyke and Leech, 1994; Pyke et al., 1994; Fitter et al., 2002). Thus, we suppose that the abnormal structure and quantity of chloroplasts might be caused by the combined impact of exceptional expression of *AaGLK* and *AaARC5*.

### The expression level of genes involved in anthocyanin biosynthesis were altered in leaf color mutants

Anthocyanin is one of the branch products in flavonoid metabolic pathway, that can be divided into six categories: delphinidin, cyanidin, pelargonidin, peonidin, petunidin and malvidin. Among these pigments, delphinidin, cyanidin and pelargonidin are the branch products of anthocyanin biosynthetic pathway (Tanaka et al., 2008). Previous research showed that *F3H* can activate the branch of pelargonidin biosynthesis and contribute to forming bright red flower in *Petunia hybrida* (Britsch and Grisebach, 1986). Our data showed that pelargonidin was the major pigment in the spathe of the *dark green*. Meanwhile the expression of *AaF3H* in the spathe of *dark green* mutant was significantly higher than that of wild type (Figure 9G). Our results demonstrated that *AaF3H* played a pivotal role in anthocyanin biosynthesis in the spathe mutant of *A. andraeanum* ‘Sonate’ and the elevated expression level of *AaF3H* could have contributed to the deep color of spathe in *dark green* mutant. It is reported that *AaDFR* plays a key role in anthocyanin biosynthesis (Vern et al., 2004). Our data showed *AaDFR* differentially expressed in distinct tissues in wild type and 4 leaf color mutants. However, the expression of *AaDFR* in *dark green* and *rubescent* mutant was generally higher than that in wild type in the same plant tissues (Figure 9E), proving that *AaDFR* is a key gene in the anthocyanin biosynthesis in *A. andraeanum* ‘Sonate.’ It is demonstrated that *DFR* contributes to pelargonidin biosynthesis because over-expression of maize *DFR* gene in petunia can produce brick-red flowers (Meyer et al., 1987). Similarly, suppression of *F3*'*5*'*H* and over-expression of DHK-reducible *DFR* resulted in pelargonidin accumulation in *Osteospermum hybrida* (Seitz et al., 2007) and petunia (Tsuda et al., 2004). In our study, the content of anthocyanin in the mutants, in particular in the spathe of the *dark green* mutant, was higher than that in the wild type (Table 3). In conclusion, the expression pattern of *AaDFR* in *A. andraeanum* is similar to that in other species. Besides genes related to anthocyanin biosynthesis, genes regulating carotenoid biosynthesis also contribute to the color phenotype in an orchid variety [*Oncidium* “Gower Ramsey”] (Hieber et al., 2006). In our study, the ratio of carotenoid/total Chl in mutants was higher than that of wild type (Table 3), suggesting that carotenoid genes may be up-regulated in the leaf color mutants.

However, the mechanism responsible for changes in the expression level of these genes is still unclear. Considering that the 4 leaf color mutants arose from tissue culture, and during the cultivation process, the *rubescent* mutant could transform into *etiolated* and *albino* mutants along with the changes of environmental and nutrient conditions, we suppose that epigenetic regulation, such as gene methylation status, might control the leaf color of mutants, which should be investigated in future study.

## Conclusions

**Figure 10:**
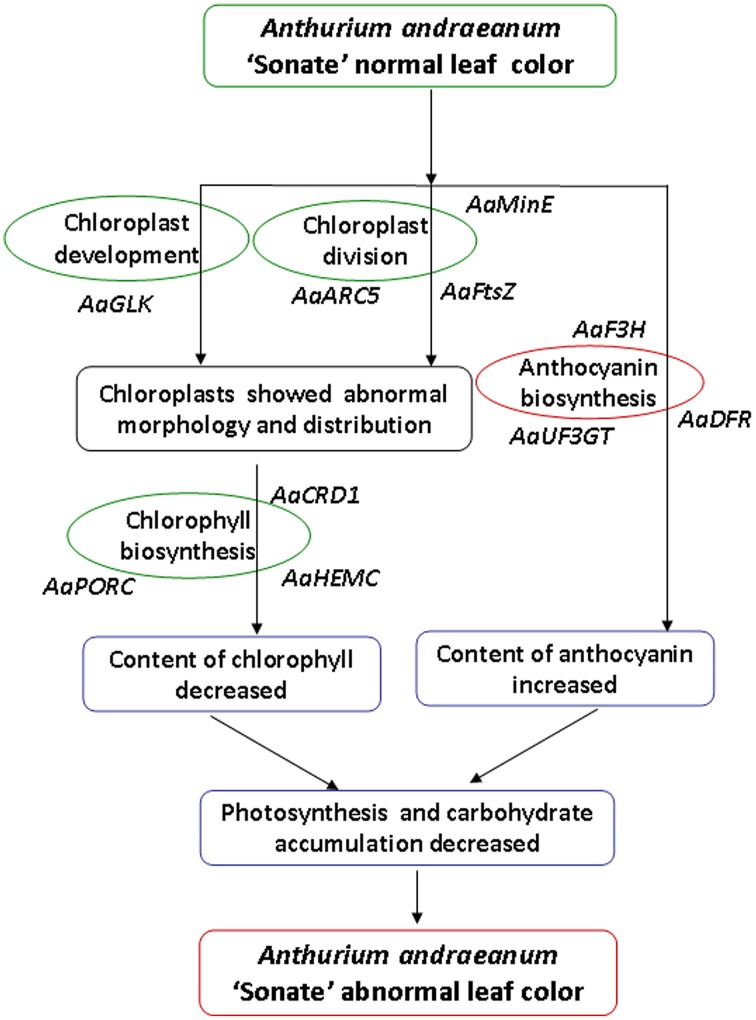
**Possible formation pathway of leaf color mutant of *Anthurium andraeanum* ‘Sonate’**.

Our analysis on the characteristics of micro- and ultra-structure and physiological characteristics showed that there were distinct differences between chloroplasts from leaf color mutants and wild type *A. andraeanum* ‘Sonate.’ Transcriptome sequence analysis has identified DEGs that are involved in chloroplast development and division, chlorophyll biosynthesis, and pigment biosynthesis and transport. qPCR experiment verified that those DEGs were differentially expressed in leaf color mutants and wild type plants. Based on results from this study, we can conclude that in leaf color mutants of *A. andraeanum* ‘Sonate,’ the abnormal chloroplast development and pigment biosynthesis directly or indirectly affected the chlorophyll and pigment biosynthesis, resulting in distinct content of chlorophyll and anthocyanin, at last forming different leaf colors (Figure 10).

### Conflict of interest statement

The authors declare that the research was conducted in the absence of any commercial or financial relationships that could be construed as a potential conflict of interest.

## Supplementary material

Figure S1 **Length distributions of contigs**.

Figure S2 **GO classification of the annotated All-unigenes and DEGs**. All terms belonged to the three main GO categories: cellular component, molecular function and biological process. The x-axis indicated the subcategories, the right y-axis indicated the number of genes in each category, the left y-axis indicated the percentage of a specific category of genes in the corresponding GO category. Purple column indicated all unigenes and red indicated DEGs.

Figure S3 **COG classification of the annotated 10,701 all-unigenes**. The capital letters on x-axis indicate the COG categories as listed on the right of the histogram and those on y-axis indicates the number of DEGs.
