# Supplementary figures and images for: Phenotype and transcriptome analysis reveals chloroplast development and pigment biosynthesis together influenced the leaf color formation in mutants of Anthurium andraeanum ‘Sonate’
Source: Front Plant Sci. 2015 Mar 11;6:139. doi: 10.3389/fpls.2015.00139 (PMC4356079; doi:10.3389/fpls.2015.00139)

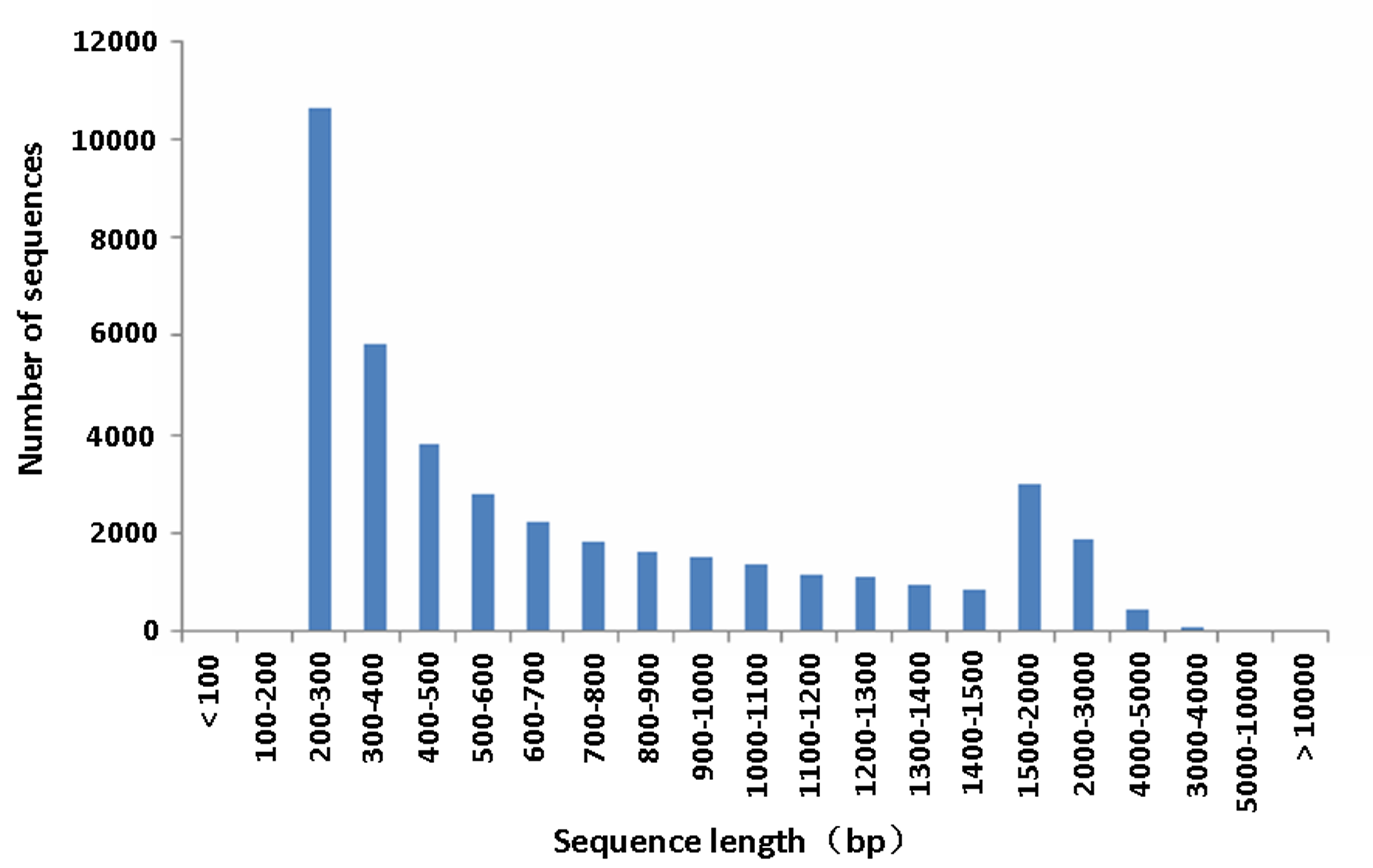

Supplement: Figure S1 — Length distributions of contigs. [file DataSheet1.ZIP › Supplementary Material/Image 1_v1.JPEG]

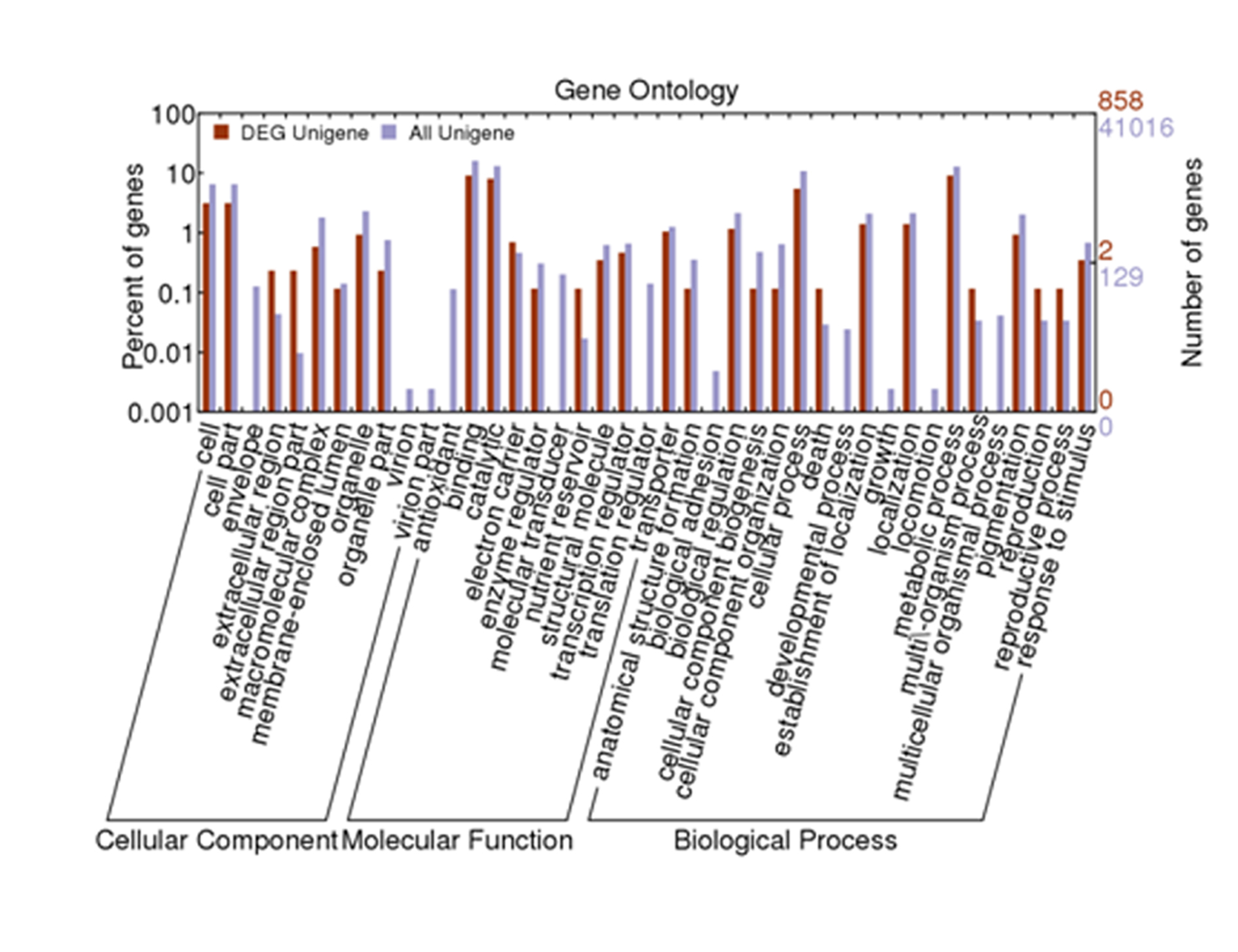

Supplement: Figure S1 — Length distributions of contigs. [file DataSheet1.ZIP › Supplementary Material/Image 2_v1.JPEG]

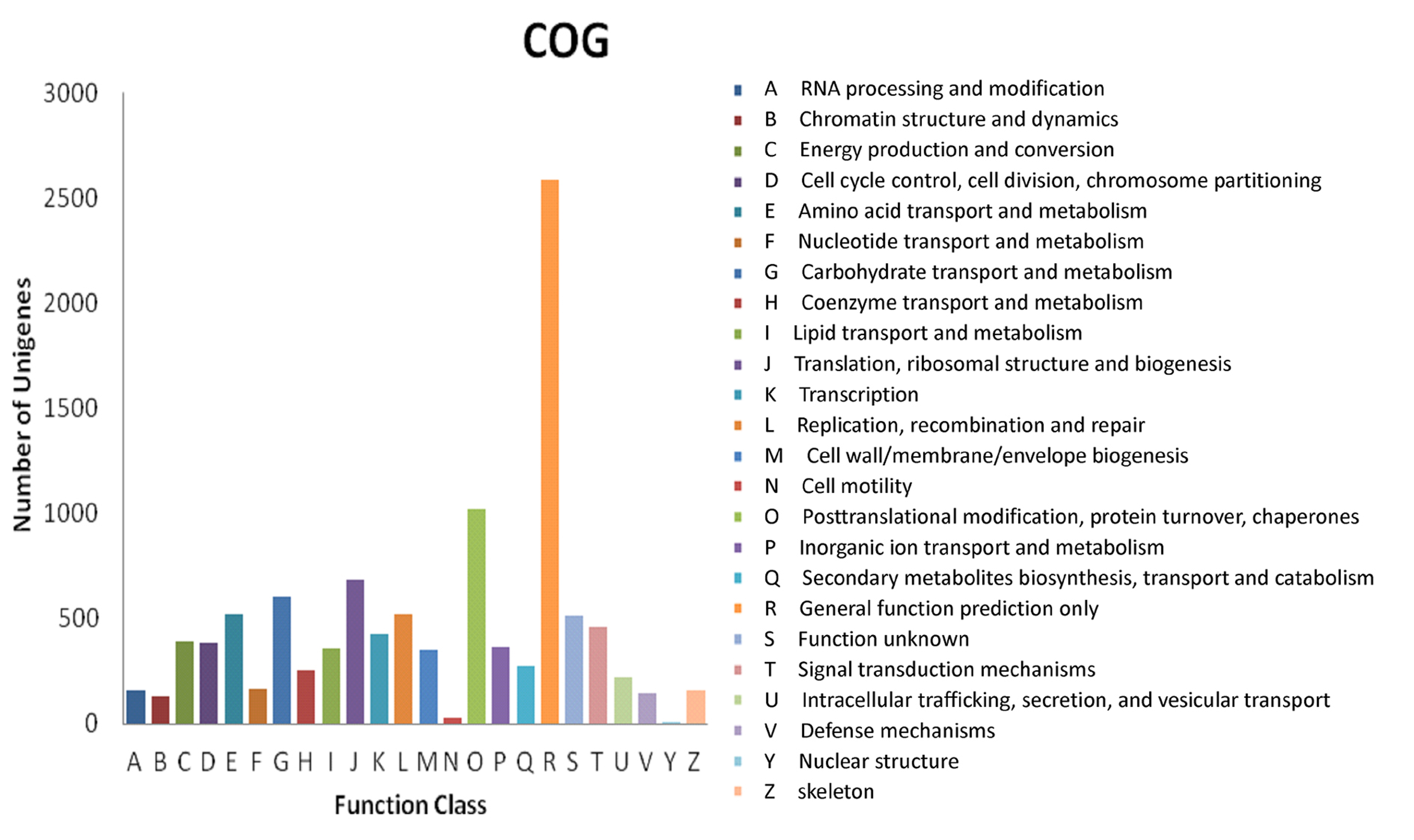

Supplement: Figure S1 — Length distributions of contigs. [file DataSheet1.ZIP › Supplementary Material/Image 3_v1.JPEG]
