# Supplementary material for: Phenotype and transcriptome analysis reveals chloroplast development and pigment biosynthesis together influenced the leaf color formation in mutants of Anthurium andraeanum ‘Sonate’
Source: Front Plant Sci. 2015 Mar 11;6:139. doi: 10.3389/fpls.2015.00139 (PMC4356079; doi:10.3389/fpls.2015.00139)
Supplement: Figure S1 — Length distributions of contigs. [file DataSheet1.ZIP › Supplementary Material/Table 1_v3.DOC]

| **Gene** | **Primer** | **Primer sequence** |
| --- | --- | --- |
| *AaGADPH* | *AaGADPH*-F | 5′- CCGAGTTCCCACTGTCGATG -3′ |
|  | *AaGADPH*-R | 5′- AATGCTCGACCTGCTGTCAC -3′ |
| *AaDFR* | *AaDFR*-F | 5′- GGATGCTGAACGTGTTGCG-3′ |
|  | AaDFR-R | 5′- AGGTCTCGTCGTAGAGGTGT-3′ |
| *AaCHS* | *AaCHS*-F | 5′- CTCTTGTGGAGGCCTTCGAG-3′ |
|  | *AaCHS*-R | 5′- ATGTTGCCGTACTCGCTCAG-3′ |
| *AaF3H* | *AaF3H-*F | 5′- GTGACCTACTTCTCGTACCCAG-3′ |
|  | *AaF3H*-R | 5′- GCACCTGGGGTAGAAGTTCA-3′ |
| *AaANS* | *AaANS*-F | 5′- CAGATCCCCACGGTGGAC-3′ |
|  | *AaANS*-R | 5′- CTGGTCGTTGGCGTACTTCT-3′ |
| *AaGLK* | *AaGLK*-F | 5′- CCACCCCATCCCATGTACA-3′ |
|  | *AaGLK*-R | 5′- CATCTCCAATGGCTGCATC-3′ |
| *AaAPRR2* | *AaAPRR2*-F | 5′- TTACCCAGTTTGGGGCCATC-3′ |
|  | *AaAPRR2*-R | 5′- GAGGCCACATTTCGCTGTTC-3′ |
| *AaMinE* | *AaMinE*-F | 5′- AGCTCAGTGTCTCGACAGAT-3′ |
|  | *AaMinE*-R | 5′- AGTCGAATGTCACATCAACAGT-3′ |
| *AaARC5* | *AaARC5*-F | 5′- AGAAGGGCCTTGCTTTCAATG-3′ |
|  | *AaARC5*-R | 5′- GGCAACTGGAGACCATCACT-3′ |
| *AaHEMC* | *AaHEMC-*F | 5′- TGCTCAAGGTGCTATTGGGA -3′ |
|  | *AaHEMC-*R | 5′- AGGCATAGCCAGCAATTGGA -3′ |
| *AaCRD1* | *AaCRD1-*F | 5′- TGAGCGGTCTTGTACTGCTG -3′ |
|  | *AaCRD1-*R | 5′- TGTTCAAGAACCCGGCATGT -3′ |
| *AaPORC* | *AaPORC-*F | 5′- CATCACCGGGAACACGAACA -3′ |
|  | *AaPORC-*R | 5′- GTGGAACTCCTGCATCGTCA -3′ |

**Table 1 Primers for qRT-PCR analysis**
