# Supplementary material for: Phenotype and transcriptome analysis reveals chloroplast development and pigment biosynthesis together influenced the leaf color formation in mutants of Anthurium andraeanum ‘Sonate’
Source: Front Plant Sci. 2015 Mar 11;6:139. doi: 10.3389/fpls.2015.00139 (PMC4356079; doi:10.3389/fpls.2015.00139)
Supplement: Figure S1 — Length distributions of contigs. [file DataSheet1.ZIP › Supplementary Material/Table 2_v3.DOC]

**Table S2 Overview of assembly quality from transcriptome in *Anthurium* *andraeanum* ‘Sonate’**

|  | Contig | Unigene |
| --- | --- | --- |
| Total number | 48,284 | 41,017 |
| Total nucleotides (nt) | 37,289,226 | 31,501,111 |
| Average length(bp) | 772 | 768 |
| Max length(bp) | 7,534 | 7,534 |
| Min length(bp) | 701 | 201 |
| GC percentage (%) | 50.30 | 50.30 |
| Length of N50 (bp) | 1,155 | 1,172 |
