# Supplementary material for: Phenotype and transcriptome analysis reveals chloroplast development and pigment biosynthesis together influenced the leaf color formation in mutants of Anthurium andraeanum ‘Sonate’
Source: Front Plant Sci. 2015 Mar 11;6:139. doi: 10.3389/fpls.2015.00139 (PMC4356079; doi:10.3389/fpls.2015.00139)
Supplement: Figure S1 — Length distributions of contigs. [file DataSheet1.ZIP › Supplementary Material/Table 3_v3.DOC]

**Table S3 Summary statistics of functional annotation for *Anthurium* *andraeanum* ‘Sonate’ leaf** unigenes in public databases

| **Public protein database** | **No. of unigene hits** | **Percentage (%)** |
| --- | --- | --- |
| Our laboratory | 41,017 | 100.0 |
| Predict protein number | 30,419 | 74.2 |
| Nr , GO, COG and KEGG | 27,539 | 67.1 |
| Nr (E-value < 10^−5^) | 25,456 | 62.1 |
| COG | 10,701 | 26.1 |
| GO | 11,593 | 28.3 |
| KEGG | 5,245 | 12.8 |
| *Arabidopsis* | 26,997 | 65.8 |
| *Oryza sativa* Japonica Group | 26,946 | 65.7 |
